# Noninvasive electrocardiographic risk factors for sudden cardiac death in dilated ca rdiomyopathy: is ambulatory electrocardiography still relevant?

**DOI:** 10.1007/s10741-023-10300-x

**Published:** 2023-03-06

**Authors:** Nikias Milaras, Panagiotis Dourvas, Ioannis Doundoulakis, Zoi Sotiriou, Vasileios Nevras, Anastasia Xintarakou, Aggeliki Laina, Stergios Soulaidopoulos, Panagiotis Zachos, Athanasios Kordalis, Petros Arsenos, Stefanos Archontakis, Christos-Konstantinos Antoniou, Dimitrios Tsiachris, Polychronis Dilaveris, Konstantinos Tsioufis, Skevos Sideris, Konstantinos Gatzoulis

**Affiliations:** 1 National and Kapodistrian University of Athens, Athens, Greece; 2 State Department of Cardiology, “Hippokration” Hospital, Vasilisis Sofias 14, 11256 Athens, Greece; 3 Department of Cardiology, General Hospital of Karditsa, Karditsa, Greece; 4 First Department of Cardiology, National and Kapodistrian University, Hippokration” Hospital, Athens, Greece; 5 Athens Heart Center, Athens Medical Center, Athens, Greece; 6 Department of Cardiology, General Hospital of Thessaloniki Gennimatas, Thessaloniki, Greece

**Keywords:** Dilated cardiomyopathy, Sudden cardiac death, Ventricular arrhythmias, Noninvasive risk factors

## Abstract

Risk stratification for sudden cardiac death in dilated cardiomyopathy is a field of constant debate, and the currently proposed criteria have been widely questioned due to their low positive and negative predictive value. In this study, we conducted a systematic review of the literature utilizing the PubMed and Cochrane library platforms, in order to gain insight about dilated cardiomyopathy and its arrhythmic risk stratification utilizing noninvasive risk markers derived mainly from 24 h electrocardiographic monitoring. The obtained articles were reviewed in order to register the various electrocardiographic noninvasive risk factors used, their prevalence, and their prognostic significance in dilated cardiomyopathy. Premature ventricular complexes, nonsustained ventricular tachycardia, late potentials on Signal averaged electrocardiography, T wave alternans, heart rate variability and deceleration capacity of the heart rate, all have both some positive and negative predictive value to identify patients in higher likelihood for ventricular arrhythmias and sudden cardiac death. Corrected QT, QT dispersion, and turbulence slope–turbulence onset of heart rate have yet to establish a predictive correlation in the literature. Although ambulatory electrocardiographic monitoring is frequently used in clinical practice in DCM patients, no single risk marker can be used for the selection of patients at high-risk for malignant ventricular arrhythmic events and sudden cardiac death who could benefit from the implantation of a defibrillator. More studies are needed in order to establish a risk score or a combination of risk factors with the purpose of selecting high-risk patients for ICD implantation in the context of primary prevention.

## Introduction

According to the World Health Organization, cardiovascular disease remains epidemiologically the most frequent cause of death worldwide [1]. It is estimated that 17 million people die each year from cardiovascular disease, and 25–50% of them die suddenly, often without having had any previous history of cardiovascular disease. Incidence of sudden cardiac death (SCD) in Europe is estimated at 1/1000 people per year, increasing with age [2–4]. Men exhibit approximately twice the risk for ACS when compared to women, but this difference decreases as age progresses [5]. With the ongoing evolution in treatment and diagnosis of the aforementioned disease, survival of patients seems to increase and SCD rates decline. According to a sub-study of the Rotterdam study, incidence of SCD in people over 40 years of age decreased from 4.7 per 1000 person-years in the 1990s to 2.1 in the 2000s [6].

### Definitions

#### Sudden cardiac death

**Fig. 1:**
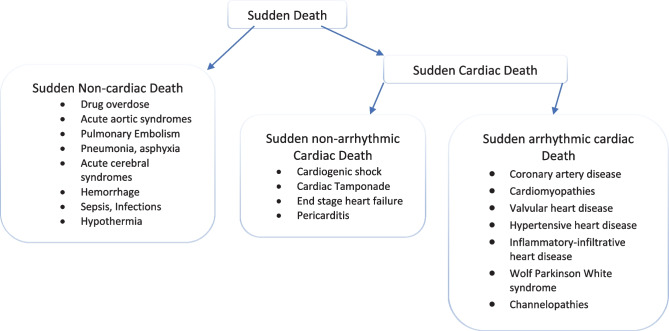
Causes of sudden death

According to the most recent definition of the World Health Organization, SCD is the unexpected death that occurs within 1 h of symptom onset (in witnessed cases) or within 24 h since the last time the person was seen alive and asymptomatic (in unwitnessed cases) [7]. SCD occurs when a trigger acts on an anatomic or functional electrophysiological substrate, with the final pathway being elicitation of ventricular fibrillation (VF) or ventricular tachycardia (VT) that degenerates into VF, causing hemodynamic collapse and cessation of mechanical activity of the heart. Asystole and electromechanical dissociation are also causes of SCD [8]. Polymorphic VT and torsade de pointes occur more frequently in patients with QT interval prolongation, either genetically inherited or drug induced. It is worth emphasizing that SCD is also caused by arrhythmic causes such as massive pulmonary embolism, aortic dissection and rupture, narcotic substances, etc. (Fig. 1). According to an older study of patients who suffered SCD while wearing a Holter monitor, VT/VF/multiform VT were the initial rhythms of cardiac arrest, with VT being the most common [9]. Electromechanical dissociation is also a common arrest rhythm, occurring mostly in noncardiac causes of death although it also commonly occurs in patients with advanced heart failure [10].

##### Dilated cardiomyopathy/nonischemic cardiomyopathy

**Fig. 2:**
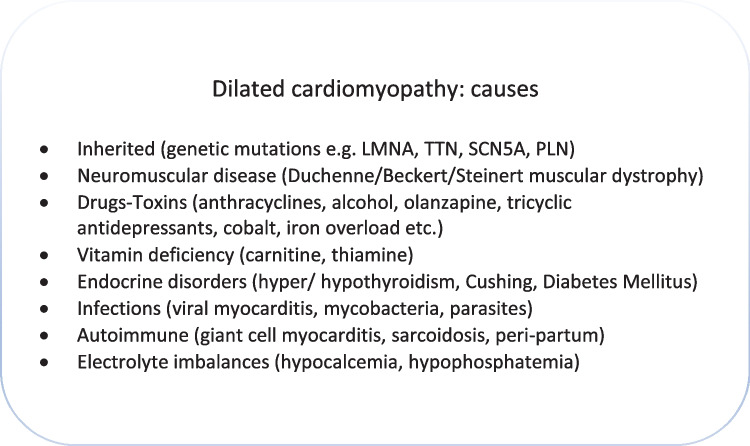
Causes of dilated cardiomyopathy

The term dilated cardiomyopathy (DCM) denotes the structural heart disease characterized by dilatation and systolic dysfunction of the left and often the right ventricle, which cannot be attributed to coronary artery disease, valvular heart disease, or arterial hypertension [11]. It is the third overall cause of heart failure in the general population and the first cause of heart transplantation worldwide [12]. DCM is an “umbrella” term as it includes numerous inherited or acquired myocardial diseases that lead to the same phenotypic outcome (e.g., myocarditis, substance and drug toxicity, autoimmune disease, gene mutations) (Fig. 2). Prevalence is estimated at 1:2500 people and is probably underestimated, as the clinical status of patients varies from asymptomatic to end-stage heart failure [13]. An often overlooked subtype of DCM is hypokinetic nondilated cardiomyopathy, characterized by left ventricular systolic dysfunction without accompanying dilatation [11]. It is underdiagnosed due to initially mild symptoms and less-pronounced imaging findings.

#### Risk stratification for sudden cardiac death

Despite the reduction in mortality that has been achieved with early diagnosis, medical therapy, and cardiovascular implantable devices, a significant percentage of patients will suffer the most dreaded complication of the disease, SCD. SCD occurs more often in young and mildly symptomatic patients. It is responsible for 30% of total DCM mortality, and 1/3 of patients are classified as functional class I according to the New York Heart Association (NYHA) classification, i.e., they are younger with mildly affected functional capacity [14]. Ventricular arrhythmias are the main cause of SCD followed by bradyarrhythmias, as myocardial fibrosis often causes conduction disturbances [15]. Apropos of VT, the main mechanism appears to be scar related re-entry followed by bundle branch re-entry [16].

Currently, primary prevention of SCD, as suggested by the European Society of Cardiology and American Heart Association guidelines is based on left ventricular ejection fraction (EF) and NYHA functional class [17, 18]. Patients qualify for an implantable cardioverter defibrillator (ICD) if their EF is below 35% and are in NYHA II-III class. The recommendations mainly considered studies such as DEFINITE, SCD-HEFT, CAT, AMIOVIRT, and COMPANION, whose patients were recruited more than 20 years ago, without receiving currently indicated pharmacotherapy. Also, several of them included a large percentage of patients with ischemic heart failure [19, 20]. Greater contribution to the level of evidence for these recommendations was given by meta-analyses of those studies, such as the one by Theuns et al. who reported a reduction of SCD along with a reduction in total mortality (RR:0.73) [21]. It is therefore reasonable to conclude that the aforementioned indicators are not characterized by sufficient sensitivity and specificity to adequately identify patients at high risk for SCD [22, 23]. Many patients with EF < 35% in whom, according to guidelines, implantation of an ICD is recommended for primary prevention of SCD, never do experience major arrhythmic events while undergoing the risks of device implantation, such as inappropriate shocks and infection. Results of the most recent DANISH study point in this direction, where ICD implantation did not reduce overall mortality in patients with DCM and low EF. The vast majority of patients were receiving optimal pharmacological and device treatment, including cardiac resynchronization devices. Patients younger than 65 years showed benefit, likely due to their fewer comorbidities, leading to the conclusion that device implantation should be reserved for those meeting multifactorial criteria beyond EF and NYHA class [24]. Conversely, an increased arrhythmic risk truly exists in some patients with EF > 35%. This was proven by SCD registries in the Oregon and Maastricht regions, in which 80% of victims had an EF that would have excluded them from ICD implantation [25, 26]. Based on the above, it is clear that the so far commonly accepted predictors of major arrhythmic events are not sufficient to unveil patients at high risk for SCD.

In the context of offering a more solid arrhythmic stratification for DCM patients, the ReCONSIDER study was conceived [27]. The ReCONSIDER study is an observational study, where imaging parameters, by cardiac magnetic resonance imaging (cMRI—such as late gadolinium enhancement) and echocardiography, along with electrocardiographic parameters (derived through 24 h Holter monitoring and signal averaged electrocardiography) are used to identify patients with EF > 35% at low or moderate risk for SCD. Those at moderate risk will undergo programmed ventricular stimulation and according to the results, they will be offered an ICD.

## Materials and methods

### Aim of the study

The purpose of this manuscript is to describe noninvasive electrocardiographic risk factors that have been studied in DCM patients, as well as their prevalence and prognostic significance in unveiling subsets at higher risk for ventricular arrhythmias and SCD.

## Inclusion criteria

(1) Studies enrolling patients aged over 18 years old, where one or more noninvasive electrocardiographic risk factors (premature ventricular complexes, nonsustained ventricular tachycardia, late potentials on signal averaged electrocardiography, T wave alternans, heart rate variability and deceleration capacity of the heart rate, corrected QT, QT dispersion and turbulence slope–turbulence onset of heart rate) were studied concerning the occurrence of VT/VF or sudden death; (2) DCM/SCD/VT were defined in accordance with the current and commonly accepted criteria [7, 11].

## Literature search strategy

**Fig. 3:**
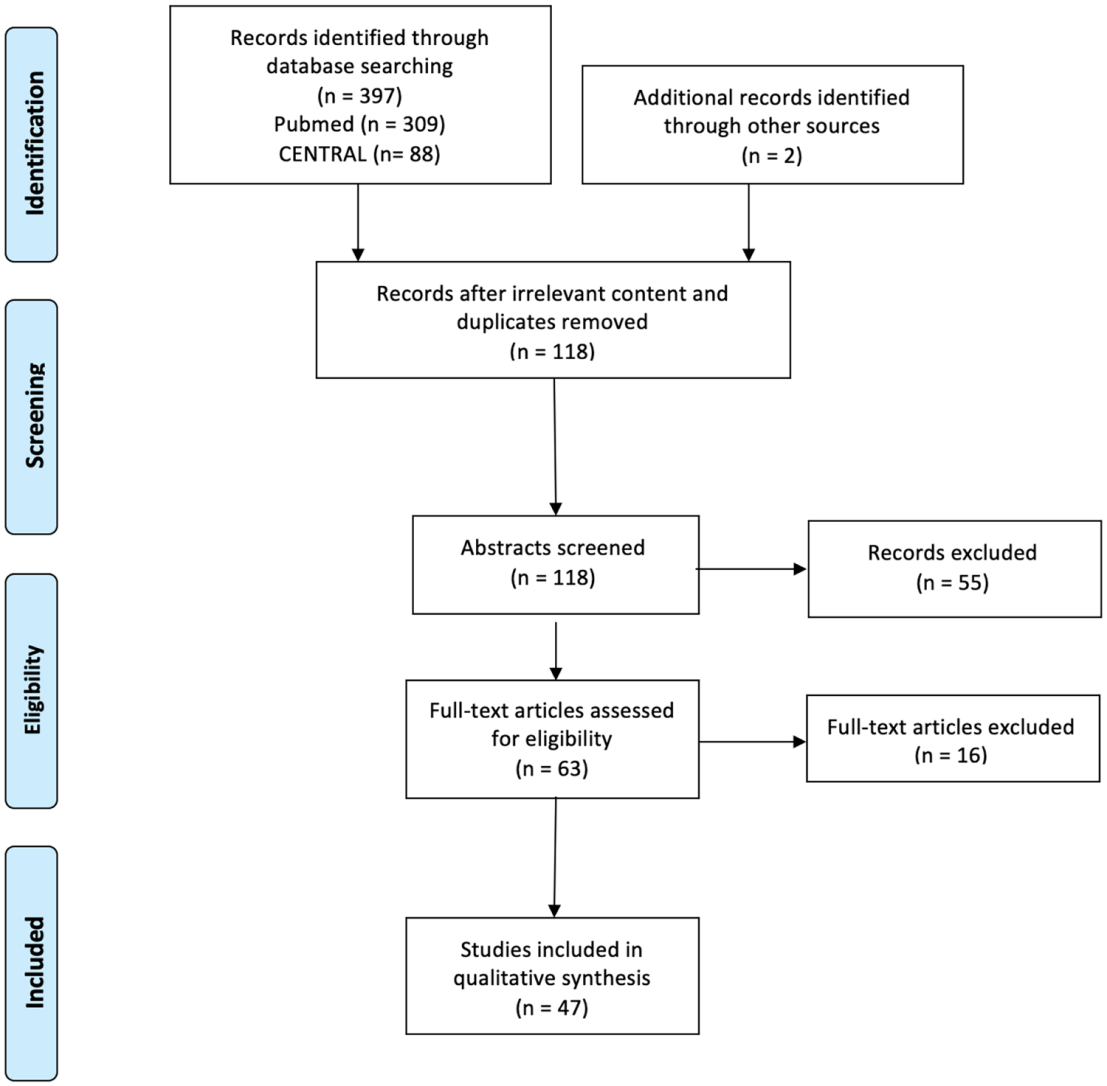
PRISMA flowchart

This systematic review and meta-analysis is reported according to the Preferred Reporting Items for Systematic reviews and Meta-Analysis (PRISMA) guidelines. We searched MEDLINE (via PubMed) and Cochrane Central Register of Controlled Trials with search terms: dilated cardiomyopathy; sudden cardiac death; ventricular arrhythmias; noninvasive risk factors (Fig. 3).

## Results

**Fig. 4:**
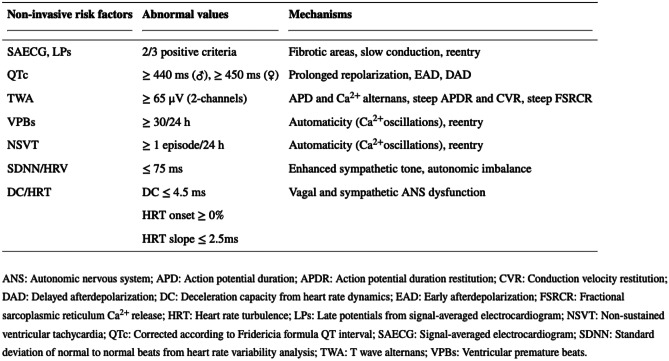
Noninvasive electrocardiographic risk factors and their correlation with mechanisms of arrhythmogenesis. Adapted with permission by P. Arsenos and Baishideng Publishing Group (World J Cardiol. 2022 Mar 26; 14(3): 139–151)

Forty-four manuscripts were finally included. Several observational studies have attempted to identify noninvasive and invasive predictors of major arrhythmic events such as late gadolinium enhancement on cardiac MRI, genetic testing, electrocardiographic testing, and programmed ventricular stimulation. In the context of this literature review, only noninvasive risk factors resulting from conventional or specialized electrocardiographic techniques are included. Each one of the arrhythmic risk markers that are going to be mentioned has been tied to at least one of the three conventional mechanisms of arrhythmogenesis: re-entry, triggered activity, and automaticity (Fig. 4).

## Microvolt TWA

T wave alternans (TWA) describes the beat-to-beat variability in time, shape, and/or amplitude of the T wave on the electrocardiogram (ECG). It is a rate-dependent phenomenon that is the result of disturbances in the management of intracellular calcium, predisposing to ventricular arrhythmias [28]. It is derived through either the spectral or the modified moving average technique. The first technique is used in treadmill stress testing while the second one allows TWA estimation in 24-h electrocardiographic recordings (Holter monitoring). It cannot be accurately measured in the presence of persistent atrial fibrillation, frequent spontaneous ventricular contractions (PVCs) and in patients with chronotropic incompetence who cannot achieve a heart rate above 110 beats per minute. Seven prospective studies with > 100 patients with DCM each examined TWA with follow-up for sudden death. In two of these, the patients were not on beta-blockers (Table 1). The common result was a high negative predictive value of TWA for sudden death, exceeding 88% in all studies [29–35]. The largest study (ALPHA) included 446 patients in whom TWA were analyzed and correlated with the endpoints of total mortality, major arrhythmic events, and sudden death. Patients with abnormal TWA were usually older, with a lower EF and advanced NYHA class. The negative predictive value was 98% for the three endpoints in a follow-up of 18 months [33].

**Table 1:** Studies assessing T wave alternans as a risk factor (in chronological order)

Name of the study or first author	DCM patients studied	Endpoint	Results (confidence interval 95%)	Year of publication
Kitamura et al. [29]	104 patients Mean FU = 21 m	SCD/VT/VF	• HR = 8.8 (1.2–65.4) • Negative predictive value = 97% • *p* = 0.0001	2002
MACAS	236 patients Mean FU = 52 m	Heart transplant free survival	• HR = 1.3 (0.59–2.90) • Negative predictive value = 90% • No statistical significance	2003
Hohnloser et al. [31]	137 patients Mean FU = 14 m	Sudden death from VT/VF	• HR = 3.44 (1.09–10.91) • Negative predictive value = 94% • *p* = 0.035	2003
Bloomfield et al. [32]	282 patients (of 587 total-51% DCM) Mean FU = 20 m	All cause mortality, nonfatal ventricular arrhythmias	• HR = 6.5% (2.4–18.1) both ischemic and DCM • Negative predictive value = 100% • *p* < 0.001	2006
ALPHA	446 patients Mean FU = 19 m	Total mortality, arrhythmic death, Lethal ventricular arrhythmias	• HR = 5.53 (1.29–23.65) • Negative predictive value = 98% • *p* = 0.004	2007
Gold et al. [34]	250 patients Mean FU = 30 m	SCD, VT/VF, appropriate ICD discharge	• HR = 1.67 (0.70–3.99) • Negative predictive value = 88% • No statistical significance	2008
Shizuta et al. [35]	264 patients (of 453 total-58% DCM) Mean FU = 36 m	SCD, VT/VF, appropriate ICD discharge	• HR = 4.43 (1.02–19.2; *p* = 0.047) both ischemic and DCM • Negative predictive value = 100% • *p* = 0.047	2012

*DCM* dilated cardiomyopathy, *FU* follow-up, *SCD* sudden cardiac death, *VT* ventricular tachycardia, *VF* ventricular fibrillation, *HR* hazard ratio, *ICD* implantable cardiac defibrillator

Based on these publications, one can assume that this risk factor may safely point towards patients with low risk for life threatening arrhythmias who may benefit little from implantation of an ICD, given its consistently high negative predictive value. The TWA study was recommended by the ISHNE guidelines in 2011 as a useful predictor of cardiovascular mortality and sudden death in patients with DCM beyond EF [36] but was excluded from their most recent guidelines in 2017 [37].

## Ventricular ectopy and nonsustained ventricular tachycardia

Premature ventricular contractions (PVCs) and nonsustained ventricular tachycardia (NSVT) are thought to arise from regions of the myocardium with increased automaticity (Table 2). It has been estimated that in 40–60% of patients with DCM NSVT is recorded while 90% show polymorphic PVCs in 24-h Holter monitoring [38]. The prognostic value of PVCs has been recognized mainly in post-infarction patients, with the presence of > 10 PVCs/h being an independent indicator of mortality [39]. At present, a study examined the presence of > 1000 PVCs/24 h in 285 patients with newly diagnosed DCM but failed to demonstrate an association with SCD/VT/VF [40]. In a subgroup analysis of the recent DANISH trial that included DCM patients with a low EF < 35% and elevated NT-ProBNP levels, the presence of > 30 PVCs per hour on Holter monitoring significantly correlated with total mortality and cardiovascular death, but not with SCD[41]. Due to the high prevalence of PVCs in this population, one cannot safely draw conclusions about their value as risk stratifiers or asses their prognostic significance. However, there are several publications about NSVT detected in 24-h electrocardiographic recordings, with often conflicting results. In the same subgroup of patients in the DANISH trial, NSVT presence was significantly associated with all-cause mortality and cardiovascular death but not with SCD. Also, in interaction analysis, no benefit would be gained from ICD implantation in these patients [41]. The most recent Leiden Nonischemic Cardiomyopathy Study demonstrated that the presence of NSVT, regardless of the presence of late gadolinium enhancement in cardiac MRI, can predict the occurrence of sustained ventricular tachyarrhythmias in patients with DCM and palpitations, out-of-hospital arrest, or a history of syncope [42]. The Marburg cardiomyopathy study highlighted 3 risk groups in patients with DCM according to the number of NSVT QRS complexes. Patients without NSVT had 2% VT/VF/SCD at 52 ± 21 months of observation, in patients with 5–9 beat NSVT runs the end point was calculated as 5%, while in those with NSVT runs consisting of more than 10 complexes, 10%. NSVT rate was not associated with the primary endpoint [43]. The multivariate analysis of a study of 319 patients with DCM by Zecchin et. al demonstrated an increased risk for SCD/VT/VF in the presence of NSVT only for patients with an EF > 35% [44]. The presence of NSVT predicted with statistical significance the total mortality and SCD even after adjustment for EF, NYHA class and age in a prospective study of 157 patients [45]. Finally, in a meta-analysis of 18 studies that examined the association of NSVT with SCD, Goldberger et al. calculated the total odds ratio to be 2.92, concluding that detection of NSVT on Holter monitoring is associated with the endpoint of SCD but is not sufficient as a single risk stratification criterion [22]. Based on the above, the presence of PVCs or NSVT is frequent in patients with DCM and this may be the reason for their only modest performance as risk stratifiers in this subset of patients.

**Table 2:** Studies assessing PVCs or NSVT as a risk factor (in chronological order)

Name of the study or first author	DCM patients studied	Endpoint	Results Confidence interval 95%	Year of publication
Becker et al. [45]	157 patients Mean FU = 22 m	Total mortality, SCD	• Total mortality (with NSVT vs without 34.2% vs 9.8%, *p* = 0.0001) • SCD (with NSVT vs without 15.8% vs 3.7%, *p* = 0.0037)	2003
Grimm et al. [43]	343 patients 3 groups 1.1.1.11..No NSVT 2.2.2.22..NSVT 3–4 complexes 3.3.3.33..NSVT 5–9 complexes 4.4.4.44..NSVT > 10 complexes Mean FU = 52 m	SCD, VT/VF	• No NSVT 2% endpoint/year • NSVT 3–9 complexes 5% endpoint/year • NSVT > 10 complexes 10% endpoint/year	2005
Fauchier et al.	162 patients Mean FU = 53 m	SCD,VT/VF	• NSVT independent predictor • *p* = 0.03	2005
Zecchin et al.[44]	319 patients Mean FU = 96 m	SCD, VT/VF, appropriate ICD shock	• EF > 35% + NSVT HR = 5.3 (1.59–17.85) • EF > 35% no NSVT HR = 0.93 (0.3–2.81) • EF < 35% similar rates regardless NSVT	2008
Spezzacatene et al. [40]	285 patients recently diagnosed > 1000 PVCs/24 h NSVT (> 5 complexes) Mean FU = 107 m	SCD, VT/VF	• HR = undefined • Negative predictive value = undefined • No statistical significance	2015
DANISH sub-study [41]	850 patients EF < 35% ­NT-ProBNP > 200 pg/mL Mean FU = 59 m	Total mortality, CVD, SCD	• PVCs > 30/h associated with total mortality HR = 1.38 (1.00–1.90), *p* = 0.046, and CVD HR = 1.78 (1.19–2.66), *p* = 0.005 • NSVT associated with total mortality HR = 1.47 (1.07–2.03), *p* = 0.02, and CVD HR = 1.89 (1.25–2.87), *p* = 0.03	2021
Piers et al. [42]	115 patients Mean FU = 48 m	VT/VF	• HR, 4.47 (1.87–10.72) • *p* = 0.001	2022

*DCM* dilated cardiomyopathy, *PVC* premature ventricular contractions, *NSVT* nonsustained ventricular tachycardia, *FU* follow-up, *CVD* cardiovascular death, *SCD* sudden cardiac death, *VT* ventricular tachycardia, *VF* ventricular fibrillation, *HR* hazard ratio, *ICD* implantable cardiac defibrillator, *EF* ejection fraction

## Late potentials on single averaged electrocardiography (Table 3)

**Table 3:** Studies assessing SAECG as a risk factor (in chronological order)

Name of the study or first author	DCM patients studied	Endpoint	Results Confidence interval 95%	Year of publication
Denereaz et al. [53]	51 patients Mean FU = 18 m	VT	Positive predictive value = 36% • Negative predictive value = 93%	1992
Marconi et al. [54]	55 DCM patients 66 controls Mean FU = 17 m	NSVT, VT	• Predicts NSVT or VT • *p* = undefined	1993
Mancini et al. [52]	114 patients Mean FU = 10 m	SCD, VT	• 1-year endpoint free for normal SAECG 95% 1-year endpoint free for abnormal SAECG 39%	1993
Turrito et al. [56]	70 patients	VT induction	• 86% predictive accuracy • *p* < 0.0003	1994
Yi et al. [57]	84 patients, spectral turbulence analysis Mean FU = 24 m	1-year survival	• Normal spectral SAECG 90% vs 63% in abnormal spectral SAECG, *p* < 0.01	1995
Goedel-Meinen et al. [51]	76 patients Mean FU = 84 m	SCD, total mortality	• 3.7-fold higher risk for SCD, *p* = 0.002 • 2.1-fold higher risk for mortality, *p* = 0.041	2001

*DCM* dilated cardiomyopathy, *FU* follow-up, *SCD* sudden cardiac death, *VT* ventricular tachycardia, *VF* ventricular fibrillation, *SAECG* signal averaged electrocardiography, *NSVT* nonsustained ventricular tachycardia

Single averaged electrocardiography (SAECG) is a method for identifying areas of slow conduction in the ventricular myocardium, which is a necessary substrate for re-entrant ventricular arrhythmias to occur (Table 3). After collection of the ECG signals, they are processed and noise is removed revealing signals in the μV range, which are not visible in the surface ECG [46]. For this test to be considered positive, at least 2 of 3 standard criteria with abnormal values must be detected. The criteria are as follows: (1) the width of the overall filtered QRS (fQRS) to be greater than 114 ms; (2) the detected electrical activity of low potentials < 40 μV within the last portion of the QRS is maintained for a period of time of at least 38 ms; (3) this electrical activity actually corresponds to a current of low potentials < 20 μV [47]. In patients with intraventricular conduction delay and QRS > 120 ms, modified criteria are applied [48]. Late potentials have been applied mainly in postmyocardial infarction patients, and their use has been declining over time [49]. Regarding DCM, positive late potentials are acquired in 25% of patients with increasing percentage in those with a history of sustained VT (70–90%) [50]. In an older study of 79 DCM patients, positive late potentials were an independent indicator for SCD (3.7-fold higher risk) and cardiac mortality (2.1-fold higher risk) [51]. These results are comparable to those reported by Mancini et al. in their study of 114 DCM patients. Two groups were created based on the presence or absence of late potentials. Sixty-six patients with negative late potentials did not suffer ventricular tachyarrhythmias or SCD in a follow-up of about 1 year. On the other hand, in 20 patients with abnormal SAECG, 4 presented with sustained VT and 5 suffered SCD. Therefore, the annual survival rate without occurrence of VT or SCD was estimated to be 95% in the absence of late potentials and only 39% in their presence [52]. A positive correlation of abnormal late potentials with the occurrence of SCD and/or VT has been detected in most relevant studies unfortunately without as high positive predictive value [53–57]. Furthermore, in a relevant meta-analysis of 10 such studies, the calculated odds ratio was 2.11 for the presence of abnormal SAECG and the primary outcome of SCD or major ventricular arrhythmic events [22]. In summary, late potentials show a correlation with SCD and VT in patients with DCM, but their use as stand-alone risk stratification tools is not recommended due to their low positive and negative predictive value [58, 59].

## QT interval and repolarization dispersion

Ventricular repolarization is considered to be a critical period of the cardiac cycle for the pathogenesis of malignant arrhythmias (Table 4). The QT interval is the measured ECG criterion regarding the duration of ventricular repolarization. Formulas have emerged that adjust the duration of this interval according to the heart rate (QTc), with the most commonly used being the Fridericia and Bazzet formulas. Prolongation of repolarization has been thoroughly investigated and tied to major arrhythmic events in ischemic cardiomyopathy, heart failure and primary “electrical” heart diseases, such as long QT syndromes [60]. The underlying mechanism of arrhythmogenesis is commonly considered to be triggered activity. In the Marburg cardiomyopathy study, QTc prolongation on surface ECG failed to reveal a tie between SCD or ventricular tachy-arrhythmias in DCM patients [30]. Of note, QTc measurement on 24-h Holter monitoring is considered to be a better reflector of this complex repolarization process when different dynamic components such as the autonomic nervous system interaction are taken into account [61]. Unfortunately, such studies could not be identified in the literature to date.

**Table 4:** Studies assessing Qt interval indices as a risk factor (in chronological order)

Name of the study or first author	DCM patients studied	Endpoint	Results Confidence interval 95%	Year of publication
Fei et al. [63]	135 patients Mean FU = 34 m	Survival, heart transplant, VT (> 3 complexes)	• QTd nonsignificant predictor	1996
Galinier et al. [65]	119 patients Mean FU = 24 m	SCD	• QTd > 80 ms RR = 4.9 (1.4–16.8) • *p* < 0.02	1998
MACAS	343 patients Mean FU = 52 m	SCD, VT/VF	• QTc on ECG and QTd nonsignificant predictors	2003
Fauchier et al. [64]	162 patients Mean FU = 53 m	SCD, VT/VF	• QTd nonsignificant predictor	2005

*DCM* dilated cardiomyopathy, *FU* follow-up, *SCD* sudden cardiac death*, VT* ventricular tachycardia, *VF* ventricular fibrillation, *QTc* corrected QT, *QTd* QT dispersion

QT dispersion (QTd) is another repolarization marker that was correlated with major arrhythmic events in various clinical settings [62]. Regarding DCM, results are conflicting. Fei et al. found no significant difference in QTd in a sample of 60 patients some of which died or received heart transplants [63]. In the aforementioned Marburg study, QTc dispersion was of no prognostic value in 343 DCM patients in regard to major arrhythmic events and SCD [30]. Same results were also reported regarding the long term risk for such endpoints by Fauchier [64]. On the other hand, in the study of Galinier et al., a QTd > 80 ms was an independent predictor of sudden death, VT, VF with correlation to other arrhythmic risk markers such as SAECG late potentials [65]. Once again, repolarization prolongation and dispersion although commonly prolonged in DCM patients, failed to predict patients in risk of worse arrhythmic outcome. It remains to be established if such repolarization indices will earn their way in assessing arrhythmic risk in DCM patients.

## Heart rate variability

Cardiovascular diseases cause disturbance in the balance of the autonomic nervous system that can predispose to ventricular arrhythmias and increased overall mortality. Whether this is due to sympathetic nervous system overload or loss of some of the activity of the parasympathetic nervous system remains unknown. Heart rate variability (HRV) quantifies the range of this variability and possibly reflects the activity of the autonomic nervous system. The classical methods of HRV analysis are (a) analysis of cardiac time series over time and (b) spectral analysis of the contained frequencies of the signal [66]. HRV is estimated in a 24-h Holter recording usually via the standard deviation of all normal RR intervals over a period of time (SDNN). HRV is implicated in numerous conditions such as arterial hypertension, diabetes mellitus, and coronary artery disease. Studies exist mainly on mixed populations with a diagnosis of heart failure, but with equivocal results (Table 5). In the literature, a meta-analysis of 4 studies, for a total of 630 patients with DCM, reported on the endpoint of SCD. Sensitivity and specificity of the index did not exceed 60% and Odds ratio was calculated as 1.72 with *p* = 0.13 [22]. Overall, the above mentioned meta-analysis did not highlight any of the autonomic nervous system assessment indices (as discussed below) as promising noninvasive risk factors for predicting death in DCM patients. In the DEFINITE trial, DCM patients with preserved HRV had an excellent prognosis on follow up and may gain little benefit from an ICD implantation. Patients were subdivided in 3 groups according to their SDNN values as SDNN < 81, 81 < SDNN < 113, SDNN > 113 with a mortality of 10%, 7%, and 0%, respectively, on a 3-year follow-up [20]. Similar results were published by Fauchier et al. where a reduced SDNN was an independent predictor of SCD as well as VT,VF (*p* = 0.01) [63]. Conversely, HRV failed to predict which patients could benefit from ICD implantation in the Marburg study [30]. Although it is now widely known that DCM patients have decreased HRV, this pathology is not formally associated with worse arrhythmic outcomes in this population and cannot serve as a risk marker for such.

**Table 5:** Studies assessing HRV as a risk factor (in chronological order)

Name of the study or first author	DCM patients studied	Endpoint	Results Confidence interval 95%	Year of publication
MACAS	343 patients Mean FU = 52 m	SCD,VT/VF	• Nonsignificant predictor	2003
Fauchier et al. [64]	162 patients Mean FU = 53 m	SCD,VT/VF	• Independent predictor • *p* = 0.01	2005
DEFINITE	274 patients Mean FU = 36 m	Total mortality	• SDNN > 113, 0% • 81 < SDNN > 113, 7% • SDNN < 81, 10% • *p* = 0.03	2006

*DCM* dilated cardiomyopathy, *FU* follow-up, *SDNN* standard deviation of NN intervals, *SCD* sudden cardiac death, *VT* ventricular tachycardia, *VF* ventricular fibrillation

## Heart rate turbulence, deceleration capacity, turbulence onset, and turbulence slope

Early PVCs in a healthy population are followed by a normal biphasic sinus node depolarization response. More specifically, each PVC is followed by a short period of acceleration followed by a period of deceleration of the heart rate. In a normal functioning autonomic nervous system, heart rate turbulence (HRT) is present and can be quantified. In cardiovascular disease, however, as previously mentioned, this turbulence is reduced or even lost. HRT is considered as an index that quantifies reflex parasympathetic activity [67]. As a risk indicator, it can be measured in a 24-h Holter recording with the assumption of the existence of a sufficient number of PVCs. HRT is quantified by turbulence onset (TO) and turbulence slope (TS) which represent the initial acceleration and subsequent deceleration of the sinus after a PVC. The phenomenon was first discovered in post myocardial infarction patients and the combination of pathological TO and TS was the strongest predictor of total mortality in the study by Schmidt et al. [68]. Regarding DCM patients, the analysis of the Marburg and Frankfurt DCM registries did not reveal a predictive value of HRT regarding major arrhythmic events [30, 69]. A meta-analysis of 3 studies by Goldberger et al. yielded similar results in 434 patients [22]. The study included the two aforementioned studies as well as a Japanese registry of patients with DCM and ischemic heart disease, in which patients with pathological HRT showed a clearly increased incidence of SCD and VT [70]. HRT was considered positive when both TO was ≥ 0% and TS was ≤ 2.5 ms/R-R interval.

Finally, deceleration capacity (DC) is thought to assess the effect of the parasympathetic nervous system on heart rate independent of sympathetic activity. Deceleration of heart rate must be the final result of the tonic sympathetic–parasympathetic interactions on the sinus node level with the reflex vagal activity to be added each moment on this tonic status [71, 72]. This is also a method of assessing the autonomic nervous system and is calculated from time series of R-R intervals in 24-h electrocardiographic monitoring. It was introduced by Bauer et al. in a study of patients after myocardial infarction [73]. Only a few studies have examined the association of this novel marker with cardiac mortality in patients with DCM. In 201 patients with a 40-month follow-up, DC below 4.5 ms powerfully and independently predicted mortality [74]. Similar results were also published by Yang, where in 65 male patients, a DC < 4.72 ms was a significant predictor of cardiac mortality on a 60-month follow-up [75]. The conclusions drawn from these studies (Table 6) are as follows: (a) patients with DCM have a reduced DC compared to their healthy counterparts and (b) a reduction in DC is strongly associated with an increase in cardiac and total mortality as well.

**Table 6:** Studies assessing HRT as a risk factor (in chronological order)

Name of the study or first author	DCM patients studied	Endpoint	Results Confidence interval 95%	Year of publication
MACAS	343 patients Mean FU = 52 m	SCD, VT/VF	• TO, TS nonsignificant predictors	2003
Klingenheben et al. [69]	114 patients Mean FU = 22 m	SCD, VT/VF	• TO, TS nonsignificant predictors	2008
Demming et al. [74]	201 patients Mean FU = 40 m	Total mortality	• DC < 4.5 ms • *p* = 0.012	2016
Yang et al. [75]	100 patients 202 healthy controls Mean FU = 60 m	Cardiac mortality	• Male patients with DC < 4.72 ms • *p* = 0.003 • Small number of female patients-analysis could not be done	2018

*DCM* dilated cardiomyopathy, *FU* follow-up, *SCD* sudden cardiac death, *VT* ventricular tachycardia, *VF* ventricular fibrillation, *TO* turbulence onset, *TS* turbulence slope, *DC* deceleration capacity

## Discussion

As previously mentioned, arrhythmia occurrence risk and sudden death risk stratification remain suboptimal in DCM. This is now widely known and proven by many investigators. On our everyday clinical practice, we encounter patients with an EF < 35% where the implantable defibrillator has never been activated, as well as patients with relatively preserved EF > 35% who present with sustained VT, VF, or suffer sudden death. Many previous and ongoing studies try to unveil a better risk stratification tool but unfortunately no single factor proved to have better sensitivity/specificity than EF. In this manner, clinicians worldwide usually rely on 24-h electrocardiographic monitoring in order to achieve a better understanding of the patients arrhythmia burden and risk. Electrocardiographic Holter monitoring can also be utilized in order to observe the therapeutic effects of antiarrhythmic agents such as beta blockers and amiodarone in heart failure. The clinical practice dissents current ISHNE guidelines on Holter monitoring, where it is stated that prognostic value is rather low and remains controversial in DCM [37]. Additionally, there is no mention of electrocardiographic monitoring in the recently published guidelines on ventricular arrhythmias and SCD of the European Society of Cardiology. Patients with an EF < 35% receive an implantable defibrillator based on a class IIa recommendation. Patients with an EF > 35% are risk layered based on 4 factors: unexplained syncope, pathogenic mutations on LMNA, PLN, FLNC, and RBM20 genes, presence of late gadolinium enhancement on cardiac MRI and inducible sustained monomorphic VT on programmed ventricular stimulation. Patients with 2 or more of these criteria qualify for primary prevention with implantation of an ICD based on a class IIa recommendation [17].

## Conclusion

Unlike ischemic cardiomyopathy, where a well-defined scar serves as a substrate for re-entrant ventricular arrhythmias and current risk stratification parameters for sudden cardiac death are adequately defined, this is not the case for DCM. Despite the scientific community’s efforts, no single risk marker stands out besides left ventricular EF which has moderate negative and positive predictive values. It remains yet unclear whether a pooling of electrocardiographic parameters or a risk score containing noninvasive risk markers can be successfully used to this end in this population.

## Data Availability

Not applicable.
